# Identification and analysis of pathogenic nsSNPs in human LSP1 gene

**DOI:** 10.6026/97320630015621

**Published:** 2019-09-30

**Authors:** Hani Mohammed Ali

**Affiliations:** 1 Department of Biological Science, Faculty of Science, King Abdulaziz University, Jeddah, Kingdom of Saudi Arabia

**Keywords:** SNP, Lymphocyte-specific protein, computational analysis, F-actin binding protein, neutrophil actin dysfunction

## Abstract

LSP1 (Lymphocyte-specific protein 1) protein plays an important role in neutrophil motility, fibrinogen matrix proteins adhesion, and trans-endothelial migration. Variation in the LSP1 gene is associated with leukemia and lymphomas in tumor cells of Hodgkin's disease and breast cancer. Despite extensive study on the human LSP1, a comprehensive analysis on the Single Nucleotide Polymorphism (SNPs) of the gene is not available. Therefore, it is of interest to identify, collect, store and analyze the SNPs of the LSP1 gene in relation to several known diseases. Hence, the SNP data (398 rsids) from dbSNP database was downloaded and mapped to the genomic coordinate of "NM_002339.2" transcript expressed by LSP1 (P33241). There were 300 nsSNPs with missense mutation in the dataset. Tools such as SIFT, PROVEAN, Condel, and PolyPhen-2 were further used to identify 29 highly deleterious or damaging on synonymous SNP (nsSNPs) for LSP1. These high confident damaging nsSNPs were further analyzed for disease association using SNPs and GO tool. SNPs of the gene such as nsSNPs C283R, G234R, Y328D and H325P showed disease association with high prevalence.

## Background

Human LSP1 (lymphocyte specific protein 1) gene encodes an intracellular F-actin binding protein, recently renamed as leukocyte specific protein. The protein is expressed in lymphocytes, macrophages, neutrophils, and endothelium and regulates adhesion to fibrinogen matrix proteins, neutrophil motility, and transendothelial migration. Due to alternative splicing there are multiple transcript variants which encodes different isoforms. Highest expression of this gene in spleen (RPKM 60.6), appendix (RPKM 43.3) and other tissues [1,2] is known. LSP1 is found in plasma membrane internal surface of the, the cytoplasm, and is thought to mediate cytoskeleton-driven responses in activated leukocytes that involve receptor capping, cell-cell interactions and cell motility [3]. Lymphocyte specific protein 1 modulates leukocyte populations in resting and inflamed peritoneum [2]. The LSP1 protein is detected in leukemia and lymphomas in tumor cells of Hodgkin's disease and breast cancer [4]. The motility of melanoma cell is inhibited even at low level of LSP1 expression [5]. Many research showed identifying the deleterious effectiveness and disease associated mutations, thus predicting the pathogenic nsSNPs in correlation to their functional and structural damaging properties [6-9]. Computational studies provide an efficient platform for analysis of genetic mutations for their pathological consequences and in determining their underlying molecular mechanism [10-11]. Single nucleotide polymorphism (SNPs) is a common genetic variations contributing greatly towards the phenotypic variations in the populations. SNPs can alter the functional consequences of proteins. In the coding region of gene, SNPs may be synonymous, non-synonymous (nsSNPs) or nonsense. Synonymous SNPs changes the nucleotide base residue but does not change the amino acid residue in protein sequence due to degeneracy of genetic code. The nsSNPs also called missense variants, alter amino acid residue in protein sequence and thus change the function of protein through altering protein activity, solubility and protein structure. Nonsense SNPs introduce premature termination in the protein sequence. SNPs have been emerged as the genetic markers for diseases and there are many SNPs markers available in the public databases. With recent advances in high-throughput sequencing technology, many new SNPs have been mapped to human LSP1genes. However, not all SNPs are functionally important. Despite extensive studies of LSP1 proteins in human and effect of their polymorphism in diseases, no attempts was made to comprehensively and systematically analyze to establish the functional consequences of SNPs of LSP1 gene. The aim of this study is to identify the high confident pathogenic SNPs of LSP1 gene and determine their functional consequences using computational methods.

## Methodology

### SNPs dataset

**Figure 1:**
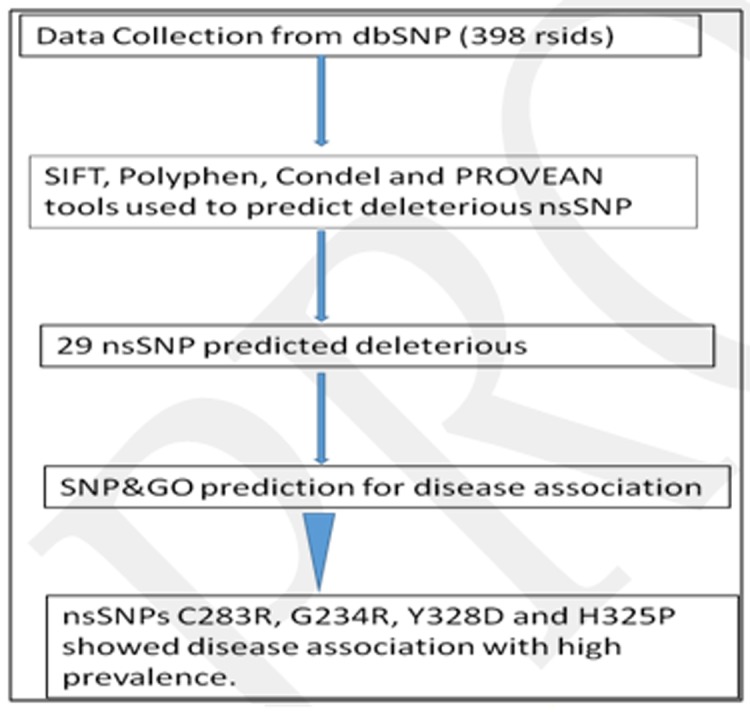
Flow chart depicting overall work methodology adopted in this study.

The SNPs of the LSP1 (Lymphocyte-specific protein 1) protein were retrieved from the dbSNP database [12]. I used "LSP1" as our search term and filter SNPs. Furthermore, I mapped these SNPs on the genomic coordinate of "NM_002339.2" transcript expresses LSP1 protein (P33241) for computation analysis of the effect of missense variant. The protein sequences of genes, LSP1 (P33241) was retrieved from the UniProt database [18]. I employed various computational approaches to identify the pathogenic SNPs and their effect on structural and functional consequences of LSP1 (Figure 1)

### Tools used for the prediction of SNPs effects Predicting deleterious and damaging nsSNPs SIFT: 

The algorithm predicted that the tolerant and intolerant coding base substitution based upon properties of amino acids and homology of sequence [13]. The tool considered that vital positions in the protein sequence have been conserved throughout evolution and therefore substitutions at conserved alignment position is expected to be less tolerated and affect protein function than those at diverse positions. I used SIFT version 2.0 [19], which predicted the amino acid substitution score from zero to one. SIFT predicted substituted amino acid as damaging at default threshold score <0.05, while score ≥ 0.05 is predicted as tolerated.

### PROVEAN:

The online tool uses an alignment-based scoring method for predicting the functional consequences of single and multiple amino acid substitutions, and in-frame deletions and insertions [14]. The tool has a default threshold score, i.e. -2.5, below which a protein variant is predicted as deleterious, and above that threshold, a protein variant is neutral.

### Condel (CONsensus DELeteriousness): 

This tool evaluates the probability of missense single nucleotide variants (SNVs) deleterious. it computes a weighted average of the scores of SIFT, PolyPhen2, Mutation Assessor and FatHMM [15].

### PolyPhen-2:

This tool is predicting the structural and functional consequences of a particular amino acid substitution in human protein [16]. Prediction of PolyPhen-2 server [20] is based on a number of features including information of structural and sequence comparison. The PolyPhen-2 score varies between 0.0 (benign) to 10.0 (damaging). The PolyPhen-2 prediction output categorizes the SNPs into three basic categories, benign (score < 0.2), possibly damaging, (score between 0.2 and0.96), or probably damaging (score >0.96).

### Predicting disease associated nsSNPsSNPs and GO:

A web server predicting whether an amino acid substitution is associated to a disease or not [17]. It is a SVM (Support Vector Machine) based tool which takes features of protein sequence, evolutionary information, and functional annotation according to Gene Ontology terms. Isoform 1 of Swiss-Prot Code of LSP1 (P33241) was used and provided the list of amino acid mutations. The results predicted the probability for the polymorphisms of helicase whether being disease-associated or not by three methods:(a) SNPs and GO, (b) PhD-SNP, and (c) PANTHER. Probability score >0.5 is predicted as disease associated variation.

## Results and Discussion:

**Figure 2:**
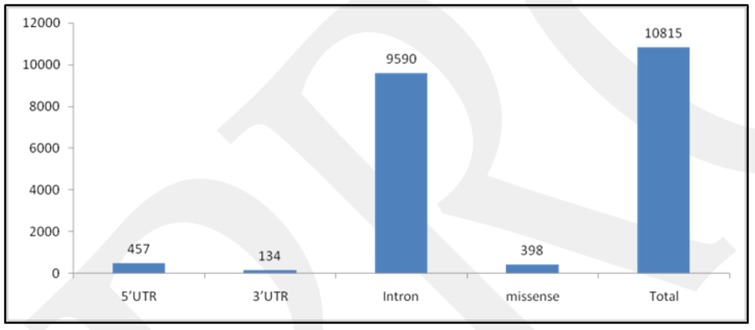
Number of SNPs in different function class of LSP1 gene of human from dbSNP database

398rsIDof nsSNPs mapped in human LSP1 gene was downloaded from dbSNP database of NCBI(Table 3), after filtering variation classSNV and function class missense,there were9590 SNPs mapped to intron, while 457SNPs mapped to 5'UTR, 134SNPs mapped to 3'UTR and 10815 mapped to total SNPs of different variation class(Figure 2). Some rsIDs are associated with multiple SNPs and therefore fall in different classes.

## Predicting deleterious and damaging nsSNPs

 In order to predict the damaging or deleteriousnsSNPs multiple consensus tools were employed. Initially,online tool VEP was used [21]. VEP advantages include: it uses latest human genome assembly GRCh38.p10, and can predict thousands of SNPs from multiple tools including SIFT, Condel, and PolyPhen-2, at a time. 398 nsSNP accession numbers were uploaded to VEP tool and the prediction results were taken for further analysis. 300 missense SNPs wasmapped to NM_002339.2 on default scores of consensus tools based on sequence and structure homology methods: (a) SIFT (score <0.5) and (b) PROVEAN (score <-2.5) and Condel(score >0.522).In order to get a very high confident nsSNPs impacting structure and function of LSP1, I considered high stringent scores across different consensus tools. At parameters of SIFT (score = 0), Polyphen(score >0.96)andCondel(score >0.9), I got 40 nsSNPs (Table 1). These 40nsSNPs were further analyzed by PROVEAN, which gave 29 nsSNP at default cutoff at -2.5 score fall in the predicted category of deleterious and havedamaging effect on protein structure and function (Table 1).

## Identifyingdisease associated nsSNPs

Furthermore, 29 selected amino acid substitutionsin LSP1 protein wereused to analyze for disease association. LSP1 Protein ID "P33241" isoform-1and its amino acid mutations were submitted to "SNPs and GO" tool [22]and the predicted disease association from three different tools were analyzed. The output of (a) SNPs and GOpredicted 4SNPsC283R, G324R, Y328D and H325Pare associated with disease and (b) PhD-SNP predicted 14 SNPsR207P, I227T, Q233R, Q233K, T235I, T235P, E239K, C283R, W297S, Y328D, Y318C, K319T, G324R,H325P are associated with diseases, while (c) PANTHER predicted 4 SNPs C283R, L296H, S276C and G301R as disease associated (Table 2).

## Conclusion

A comprehensive analysis of SNPs of the human LSP1protein with known disease-associatedmutations is reported for the first time. The study identified 29 nsSNPs as highly damaging nsSNPsof the human LSP1protein. These high confident damaging nsSNPs were further analyzed fordisease association by manual data mapping. Prediction analysis showsthat SNPs C283R, G324Rand H325P and Y328D have high prevalence for disease association. Data implies that the reportednsSNPs could potentially alter structure and hence the function of LSP1 protein resulting inpathogenicity with abnormal symptoms describing the disease states. These nsSNPs were associatedwith significant pathogenicity pending experiment verification to link disease prevalence.

## Figures and Tables

**Table 1:** List of 40 deleterious missense SNPs on the LSP1 gene identified using prediction tools such as SIFT (score = 0),Condel (score >0.9),Polyphen (score >0.96)and PROVEAN (score =-2.5).

SNP ids	AA Change	SIFT (score)	Polyphen (score)	Condel (score)	PROVEAN
rs757274538	E74Q	deleterious(0)	probably_damaging(0.924)	deleterious(0.818)	Neutral
rs1427708683	D78N	deleterious(0)	probably_damaging(0.932)	deleterious(0.823)	Deleterious
rs371381465	E79K	deleterious(0)	probably_damaging(0.934)	deleterious(0.825)	Neutral
rs371381465	E79Q	deleterious(0)	probably_damaging(0.946)	deleterious(0.835)	Neutral
rs767014224	S177N	deleterious(0)	probably_damaging(0.961)	deleterious(0.849)	Neutral
rs148262402	D200Y	deleterious(0)	probably_damaging(0.963)	deleterious(0.850)	Deleterious
rs764746759	R207P	deleterious(0)	probably_damaging(0.963)	deleterious(0.850)	Deleterious
rs1347663065	S212R	deleterious(0)	probably_damaging(0.963)	deleterious(0.850)	Deleterious
rs1172211080	S214R	deleterious(0)	probably_damaging(0.972)	deleterious(0.859)	Deleterious
rs1225441968	Q219H	deleterious(0)	probably_damaging(0.973)	deleterious(0.859)	Neutral
rs1321265627	L222S	deleterious(0)	probably_damaging(0.977)	deleterious(0.863)	Neutral
rs1223328434	P223R	deleterious(0)	probably_damaging(0.977)	deleterious(0.863)	Deleterious
rs1482882164	S225F	deleterious(0)	probably_damaging(0.977)	deleterious(0.863)	Deleterious
rs375066461	I227V	deleterious(0)	probably_damaging(0.98)	deleterious(0.869)	Neutral
rs746869893	I227T	deleterious(0)	probably_damaging(0.984)	deleterious(0.875)	Deleterious
rs769418125	E232G	deleterious(0)	probably_damaging(0.985)	deleterious(0.877)	Deleterious
rs1163688948	Q233K	deleterious(0)	probably_damaging(0.987)	deleterious(0.881)	Deleterious
rs1366846876	Q233R	deleterious(0)	probably_damaging(0.99)	deleterious(0.886)	Deleterious
rs748573553	T235I	deleterious(0)	probably_damaging(0.99)	deleterious(0.886)	Deleterious
rs775207068	T235P	deleterious(0)	probably_damaging(0.99)	deleterious(0.886)	Deleterious
rs375475958	E239K	deleterious(0)	probably_damaging(0.991)	deleterious(0.889)	Deleterious
rs767390484	R249S	deleterious(0)	probably_damaging(0.992)	deleterious(0.892)	Deleterious
rs1392782919	T263N	deleterious(0)	probably_damaging(0.994)	deleterious(0.897)	Deleterious
rs771463495	T269R	deleterious(0)	probably_damaging(0.995)	deleterious(0.902)	Deleterious
rs1263005551	S276Y	deleterious(0)	probably_damaging(0.995)	deleterious(0.902)	Deleterious
rs1263005551	S276C	deleterious(0)	probably_damaging(0.996)	deleterious(0.906)	Deleterious
rs760554324	C283R	deleterious(0)	probably_damaging(0.996)	deleterious(0.906)	Deleterious
rs1327088229	L296H	deleterious(0)	probably_damaging(0.996)	deleterious(0.906)	Deleterious
rs757906951	W297S	deleterious(0)	probably_damaging(0.997)	deleterious(0.911)	Deleterious
rs767954738	E298K	deleterious(0)	probably_damaging(0.997)	deleterious(0.911)	Neutral
rs1203026216	G301R	deleterious(0)	probably_damaging(0.998)	deleterious(0.919)	Deleterious
rs556754848	G315R	deleterious(0)	probably_damaging(0.998)	deleterious(0.919)	Deleterious
rs1345247398	K316Q	deleterious(0)	probably_damaging(0.998)	deleterious(0.919)	Neutral
rs974685665	Y318C	deleterious(0)	probably_damaging(0.998)	deleterious(0.919)	Deleterious
rs758730712	K319T	deleterious(0)	probably_damaging(0.998)	deleterious(0.919)	Deleterious
rs578141909	V321L	deleterious(0)	probably_damaging(0.998)	deleterious(0.919)	Neutral
rs1490256278	V321A	deleterious(0)	probably_damaging(0.999)	deleterious(0.935)	Neutral
rs745616898	G324R	deleterious(0)	probably damaging(0.999)	deleterious(0.935)	Deleterious
rs1468912408	H325P	deleterious(0)	probably damaging(0.999)	deleterious(0.935)	Deleterious
rs1409361986	Y328D	deleterious(0)	probably_damaging(0.999)	deleterious(0.935)	Deleterious

**Table 2:** Prediction of disease associated amino acid substitution using SNPs and GO, PhD-SNP and PNTHER on29 deleterious or damaging missense SNP using tools such as SIFT, Condel, Polyphen and PROVEAN

SNP ids	AA Change	PhD-SNP	PANTHER	SNPs and GO
rs1427708683	D78N	Neutral	Unclassified	Neutral
rs148262402	D200Y	Neutral	Unclassified	Neutral
rs764746759	R207P	Disease	Unclassified	Neutral
rs1347663065	S212R	Neutral	Unclassified	Neutral
rs1172211080	S214R	Neutral	Unclassified	Neutral
rs1223328434	P223R	Neutral	Unclassified	Neutral
rs1482882164	S225F	Neutral	Unclassified	Neutral
rs746869893	I227T	Disease	Unclassified	Neutral
rs769418125	E232G	Neutral	Unclassified	Neutral
rs1163688948	Q233K	Disease	Unclassified	Neutral
rs1366846876	Q233R	Disease	Unclassified	Neutral
rs748573553	T235I	Disease	Unclassified	Neutral
rs775207068	T235P	Disease	Unclassified	Neutral
rs375475958	E239K	Disease	Unclassified	Neutral
rs767390484	R249S	Neutral	Neutral	Neutral
rs1392782919	T263N	Neutral	Neutral	Neutral
rs771463495	T269R	Neutral	Neutral	Neutral
rs1263005551	S276Y	Neutral	Neutral	Neutral
rs1263005551	S276C	Neutral	Disease	Neutral
rs760554324	C283R	Disease	Disease	Disease
rs1327088229	L296H	Neutral	Disease	Neutral
rs757906951	W297S	Disease	Neutral	Neutral
rs1203026216	G301R	Neutral	Disease	Neutral
rs556754848	G315R	Neutral	Unclassified	Neutral
rs974685665	Y318C	Disease	Unclassified	Neutral
rs758730712	K319T	Disease	Unclassified	Neutral
rs745616898	G324R	Disease	Unclassified	Disease
rs1468912408	H325P	Disease	Unclassified	Disease
rs1409361986	Y328D	Disease	Unclassified	Disease

**Table 3:** List of 398 missense SNPs rs idsof human LSP1

rs621679	rs565801400	rs772183681	rs1202288341	rs1366951082
rs1140212	rs567011070	rs773812500	rs1203026216	rs1367148625
rs1803928	rs569184113	rs774174728	rs1206197758	rs1371307311
rs7929248	rs570838125	rs774187451	rs1206383331	rs1373484177
rs7938342	rs573166009	rs774759615	rs1208571311	rs1377557151
rs11545725	rs574262123	rs775207068	rs1209026745	rs1381324548
rs57352451	rs574587041	rs775690374	rs1211172432	rs1381440832
rs138247091	rs575334014	rs775783036	rs1213020747	rs1385778938
rs138303369	rs576282068	rs775796745	rs1214643505	rs1390296970
rs138504655	rs577178834	rs777162986	rs1218116157	rs1390700870
rs140673005	rs578141909	rs777226710	rs1222043175	rs1391914838
rs141664313	rs745616898	rs777617464	rs1223328434	rs1392782919
rs141902712	rs746345460	rs778193946	rs1224210148	rs1394437978
rs142354742	rs746869893	rs778252754	rs1225441968	rs1396783838
rs144778074	rs747106345	rs779033742	rs1226157177	rs1399794061
rs144840874	rs747369818	rs779711392	rs1227502672	rs1407439097
rs145216198	rs747468057	rs779796182	rs1232033724	rs1409361986
rs146468121	rs747544389	rs779888159	rs1233355677	rs1410605938
rs147310705	rs747621569	rs780821356	rs1234696950	rs1412542490
rs147890004	rs747742064	rs781120168	rs1240434942	rs1413977301
rs147990493	rs748208610	rs781492964	rs1241527965	rs1414831389
rs148042410	rs748401091	rs866361186	rs1242184369	rs1416863114
rs148262402	rs748573553	rs866872817	rs1243095899	rs1417172855
rs148966414	rs749677355	rs866926158	rs1243463270	rs1420092778
rs149086047	rs750149067	rs867314806	rs1243674302	rs1422008007
rs149491406	rs750915233	rs868173065	rs1245841526	rs1422217064
rs150432651	rs750992011	rs868500426	rs1247334626	rs1423047689
rs150456040	rs751107694	rs878889192	rs1247536599	rs1423202063
rs150542237	rs751527292	rs879106981	rs1249156883	rs1427708683
rs181774507	rs752408075	rs887699875	rs1250098025	rs1430559453
rs182693925	rs753356088	rs888898118	rs1250264665	rs1430649392
rs184276196	rs753582906	rs891974211	rs1250725212	rs1434072090
rs189506078	rs754249948	rs892720144	rs1251749609	rs1435814360
rs199756727	rs754745738	rs895629191	rs1254008276	rs1437946454
rs199783035	rs755253787	rs904377789	rs1261074251	rs1441513398
rs200019612	rs755491188	rs910560883	rs1263005551	rs1445305286
rs200067113	rs755782795	rs918757420	rs1265743121	rs1446623366
rs200522804	rs756566635	rs923411713	rs1267291484	rs1446638347
rs200748215	rs757274538	rs927406502	rs1270611893	rs1448500435
rs201040841	rs757527171	rs945925029	rs1270942861	rs1448961397
rs201670929	rs757725608	rs945952465	rs1274339078	rs1449103483
rs202240419	rs757906951	rs948939081	rs1282238877	rs1450439364
rs267602812	rs758125057	rs952815816	rs1282961531	rs1452607509
rs368052660	rs758730712	rs952911063	rs1285324855	rs1452916657
rs368065769	rs759191270	rs959933771	rs1291913683	rs1453014034
rs368886999	rs760171733	rs968424839	rs1293971450	rs1455071304
rs369531651	rs760554324	rs974685665	rs1294666770	rs1457081847
rs369993990	rs760660868	rs986034440	rs1295737723	rs1460940380
rs370562045	rs761235196	rs998239117	rs1298324143	rs1462548468
rs370626038	rs762396204	rs1000519442	rs1299382126	rs1464317130
rs371381465	rs762776917	rs1007609338	rs1299426387	rs1465757982
rs371615334	rs762959419	rs1008657306	rs1303610213	rs1466007447
rs372030914	rs763868652	rs1025737858	rs1305747114	rs1467909235
rs372146610	rs763948767	rs1025876594	rs1313911503	rs1468535644
rs372450003	rs764143258	rs1029191221	rs1314295624	rs1468912408
rs373309025	rs764725057	rs1030510358	rs1318719888	rs1471227409
rs373401268	rs764746759	rs1033571885	rs1321122730	rs1473317251
rs373858328	rs764816812	rs1044635385	rs1321265627	rs1474105982
rs374406652	rs764861687	rs1053321895	rs1323838117	rs1475328014
rs375066461	rs765067269	rs1158055638	rs1327088229	rs1475667216
rs375326868	rs765491711	rs1158576776	rs1332594197	rs1479358037
rs375475958	rs766377117	rs1159958990	rs1333563092	rs1482882164
rs376029050	rs766651093	rs1163688948	rs1334846403	rs1484168260
rs376300668	rs766827824	rs1168844856	rs1335833290	rs1484297408
rs376328301	rs766836969	rs1169358177	rs1336103012	rs1486073931
rs527743009	rs767014224	rs1170512001	rs1337630668	rs1490256278
rs530862911	rs767061907	rs1172211080	rs1339506361	rs1490261047
rs534563533	rs767390484	rs1173604116	rs1340203839	rs1565074079
rs535919851	rs767954738	rs1177125352	rs1344481079	rs1565074311
rs538542793	rs768294917	rs1180876266	rs1345247398	rs1565085055
rs539714151	rs768625571	rs1184872981	rs1347018258	rs1565085108
rs545999529	rs769418125	rs1186423669	rs1347663065	rs1565086802
rs553028792	rs769962820	rs1187059148	rs1349693890	rs57739592
rs556754848	rs770047466	rs1189732756	rs1349980392	rs17855362
rs557026040	rs770153360	rs1192423892	rs1357448958	rs16927670
rs558867326	rs770329540	rs1193486906	rs1358207243	rs3188464
rs561026287	rs770351321	rs1194011645	rs1358988213	rs186236551
rs563563171	rs771463495	rs1194094051	rs1361728086	rs57757026
rs564056573	rs771507322	rs1196917140	rs1366398226	
rs564198572	rs772024277	rs1201337942	rs1366846876
